# Improved Alkaline Hydrogen Evolution Performance of Dealloying Fe_75−x_Co_x_Si_12.5_B_12.5_ Electrocatalyst

**DOI:** 10.3390/molecules29174130

**Published:** 2024-08-30

**Authors:** Si-Cheng Zhong, Zhe Cui, Jia Li, Guang-Run Tian, Zhong-Hong Zhou, Hong-Fei Jiao, Jie-Fu Xiong, Li-Chen Wang, Jun Xiang, Fu-Fa Wu, Rong-Da Zhao

**Affiliations:** 1 School of Materials Science and Engineering, Liaoning University of Technology, Jinzhou 121001, China; 2 School of Material Science and Engineering, China University of Mining and Technology, Xuzhou 221008, China; 3 Innovation Center for Applied Magnetics Co., Ltd., Ningbo 315201, China; 4 Ningbo Institute of Materials Technology and Engineering, Chinese Academy of Sciences, Ningbo 315201, China

**Keywords:** dealloying, Fe-based compounds, doping, hydrogen evolution reaction, electrocatalytic

## Abstract

The electrocatalytic performance of a Fe_65_Co_10_Si_12.5_B_12.5_ Fe-based compounds toward alkaline hydrogen evolution reaction (HER) is enhanced by dealloying. The dealloying process produced a large number of nanosheets on the surface of NS-Fe_65_Co_10_Si_12.5_B_12.5_, which greatly increased the specific surface area of the electrode. When the dealloying time is 3 h, the overpotential of NS-Fe_65_Co_10_Si_12.5_B_12.5_ is only 175.1 mV at 1.0 M KOH and 10 mA cm^−2^, while under the same conditions, the overpotential of Fe_65_Co_10_Si_12.5_B_12.5_ is 215 mV, which is reduced. In addition, dealloying treated electrodes also show better HER performance than un-dealloying treated electrodes. With the increase in Co doping amount, the overpotential of the hydrogen evolution reaction decreases, and the hydrogen evolution activity is the best when the addition amount of Co is 10%. This work not only provides a basic understanding of the relationship between surface activity and the dealloying of HER catalysts, but also paves a new way for doping transition metal elements in Fe-based electrocatalysts working in alkaline media.

## 1. Introduction

In the face of the excessive use of traditional fossil energy and the increasingly serious problem of environmental pollution, the urgent search for efficient, environmentally friendly and sustainable new energy has become an urgent task for researchers [1,2]. Hydrogen has attracted much attention as an ideal alternative energy source to solve pollution problems and energy crises [3,4]. At present, the most advanced hydrogen evolution electrocatalyst is the Pt-based [5,6,7,8,9,10,11] nanomaterial catalyst, but its cost, scarcity, and difficult storage bring great limitations, which hinder the production of hydrogen by electrolytic water [12,13,14,15,16,17]. Therefore, it is very important in the design and synthesis of stable, low-cost and high-efficiency hydrogen evolution catalysts. Element doping is an important research direction in the field of electrocatalysis [18,19,20,21]. Researchers have tried to improve the performance of the catalyst by doping other elements. The purpose of doping is to change the electronic structure of the catalyst, increase the number of active sites and intrinsic activity, or improve its stability [1,19,22].

Fe-based compounds have a high electrochemically active surface area, and more importantly, the synergistic effect between metals and nonmetals can expand the contact area between a catalyst and electrolyte, and can also promote gas adsorption and desorption [23]. Therefore, Fe-based compounds have received extensive attention in the fields of hydrogen storage and electrocatalysis. At present, dealloying corrosion is the most commonly used method for metal surface modification, which can selectively remove one or more elements to form a nanoporous structure and a structure with a large electrochemically active area, thereby improving electrocatalytic activity [3,24,25,26]. At present, the electrocatalytic hydrogen evolution of Fe-based compounds is studied systematically, including FeSiB Fe-based compounds. The electrical conductivity and operation durability of Fe are good, coupled with the strong interfacial interaction between Si and B elements, the non-metallic elements can be removed after dealloying corrosion, thus modifying the metal surface, exposing a large area of the active site on the surface of the catalyst, and further improving the electrocatalytic activity [27].

In the design of nanocomposite catalysts, the porous structure can bring about chemical synergies between components, thus showing excellent electrocatalytic performance beyond that of a single component [3]. By carefully arranging atoms or molecules at the hybrid interface, changes in electronic or chemical properties can be triggered, activating synergies and greatly enhancing the efficiency of electrocatalysis [28]. At the same time, the introduction of different transition metals (such as Co) can significantly change the nanostructure, catalytic efficiency, and mechanism of action of metal-based catalysts. Doped Co can not only effectively improve the electrocatalytic activity by adjusting the electronic structure, but also improve the adsorption free energy of hydrogen and the ability to adsorb and dissociate water [29].

For example, Zhu et al. used a simple one-step hydrothermal method to synthesize cobalt-doped vanadium diselenide (VSe_2_) nanosheets, and the catalytic activity of Co-doped VSe_2_ in a hydrogen evolution reaction in acidic solution was significantly enhanced, because Co doping significantly reduced the Gibbs free energy of hydrogen adsorption and promoted electron transfer and hydrogen evolution reaction kinetics [30]. Fomekong et al. improved the electrocatalytic performance by introducing Co into the precursor and ZnO framework, which greatly reduced the energy band gap and increased the number of defects in the structure. The optimal amount of Co leads to the abundance of defects and the reduction in the band gap in the prepared materials. With the increase in defects, the increase in defects and active sites, and the improvement of charge transfer due to the reduction in the band gap, Co-ZnO has high hydrogen evolution activity in alkaline media [31].

In this paper, the catalyst Fe_65_Co_10_Si_12.5_B_12.5_ was prepared by arc melting. The effects of Co_x_ doping on the microstructure of Fe_75−x_Co_x_Si_12.5_B_12.5_ catalyst and the performance of hydrogen evolution reaction were studied. The changes in the microstructure and HER properties of Fe_75−x_Co_x_Si_12.5_B_12.5_ catalyst and dealloying corrosion treatment catalyst were compared.

## 2. Results and Discussion

### 2.1. Structure Characterization

**Figure 1:**
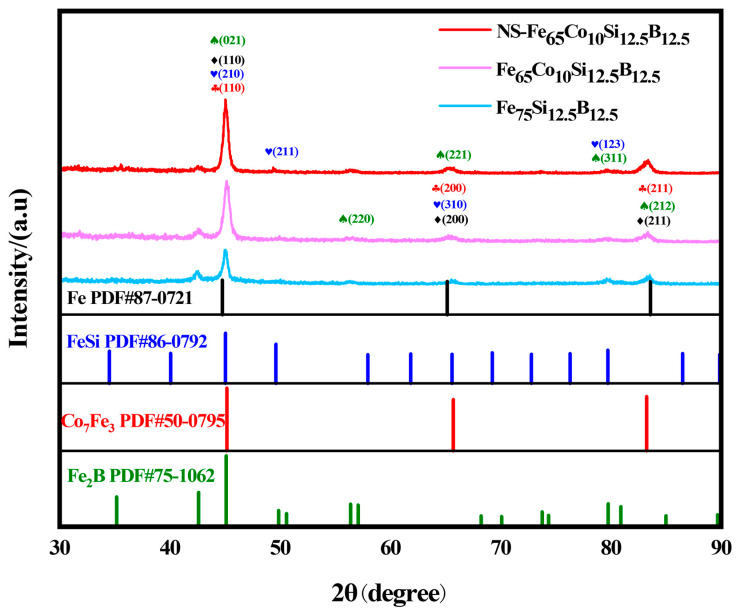
X-ray diffraction patterns of NS-Fe_65_Co_10_Si_12.5_B_12.5_, Fe_65_Co_10_Si_12.5_B_12.5_, and Fe_75_Si_12.5_B_12.5_ electrodes.

Figure 1 shows the X-ray diffraction patterns of Fe_65_Co_10_Si_12.5_B_12.5_ and Fe_75_Si_12.5_B_12.5_ catalysts with different treatments. Among them, the red diamond represents the sample of NS-Fe_65_Co_10_Si_12.5_B_12.5_. The pink diamond represents the sample of Fe_65_Co_10_Si_12.5_B_12.5_. The blue diamond represents the sample of Fe_75_Si_12.5_B_12.5_. As shown in the X-ray diffraction patterns of Fe_75_Si_12.5_B_12.5_ and Fe_65_Co_10_Si_12.5_B_12.5_ in Figure 1, peaks with 2θ angles of 44.675°, 65.026°, and 82.339° correspond to the crystal faces of Fe (110), (200), and (211), respectively. Peaks with 2θ angles of 45.047°, 65.603°, and 79.730° correspond to (210), (310), and (123) crystal faces of FeSi, respectively. The 2θ peaks of 42.611°, 45.107°, 56.339°, and 79.763° correspond to the (002), (211), (202), and (330) crystal faces of Fe_2_B, respectively. The diffraction peaks of FeSi and Fe_2_B are consistent with those of Fe_75_Si_12.5_B_12.5_, Fe_65_Co_10_Si_12.5_B_12.5_, and NS-Fe_65_Co_10_Si_12.5_B_12.5_ The peaks of Co_7_Fe_3_ at 2θ angles of 45.169°, 65.712°, and 83.252° are (110), (200), and (211), respectively. This proved that the prepared Fe_65_Co_10_Si_12.5_B_12.5_ of different treatments contained Fe, Si, B, and Co, corresponding to EDS energy spectrum, and that the NS-Fe_65_Co_10_Si_12.5_B_12.5_ catalyst was successfully synthesized.

Compared with Fe_75_Si_12.5_B_12.5_, Fe_65_Co_10_Si_12.5_B_12.5_ has a stronger diffraction intensity, indicating that Co doping can effectively improve the crystallinity of the material. In particular, the increase in the crystallinity of the Fe-Si phase reflects the enhanced binding effect of Si and metal. After dealloying NS-Fe_65_Co_10_Si_12.5_B_12.5_, the Fe-Si phase disappeared and the Fe phase appeared, indicating that Si was dissolved in an alkaline solution during the dealloying treatment. The intensity of the 42.611° diffraction peak is weakened compared with the unetched one, indicating that part of the B element is dissolved in the process of dealloying corrosion.

### 2.2. Morphology Characterization

**Figure 2:**
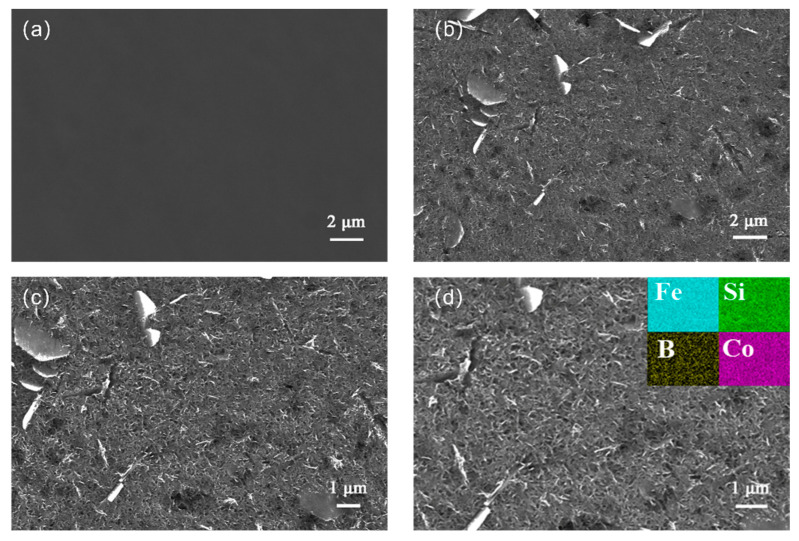
Scanning electron microscope images of Fe_65_Co_10_Si_12.5_B_12.5_ and NS−Fe_65_Co_10_Si_12.5_B_12.5_ for different magnifications. (**a**) SEM images of the Fe_65_Co_10_Si_12.5_B_12.5_. (**b**–**d**) SEM images of NS-Fe_65_Co_10_Si_12.5_B_12.5_ at 5000 times magnification (**b**), 7000 times magnification (**c**), and 8000 times magnification (**d**).

The surface morphology of Fe_65_Co_10_Si_12.5_B_12.5_ before and after dealloying was observed by a scanning electron microscope. Figure 2 shows the scanning electron microscope images of Fe_65_Co_10_Si_12.5_B_12.5_ and NS-Fe_65_Co_10_Si_12.5_B_12.5_. Figure 2a,b shows the surface images of Fe_65_Co_10_Si_12.5_B_12.5_ and NS-Fe_65_Co_10_Si_12.5_B_12.5_ magnified by 5000 times. After corrosion, a nanosheet structure was formed on the surface of NS-Fe_65_Co_10_Si_12.5_B_12.5_. Figure 2c shows a 7000-fold scanning electron microscope image of NS-Fe_65_Co_10_Si_12.5_B_12.5_. Figure 2d shows the energy dispersive X-ray spectrum image of NS-Fe_65_Co_10_Si_12.5_B_12.5_. It can be clearly seen from the figure that the content distribution of Fe, Si, B, and Co is consistent with the results of the X-ray diffraction spectrum [30].

### 2.3. Chemical State Analysis

**Figure 3:**
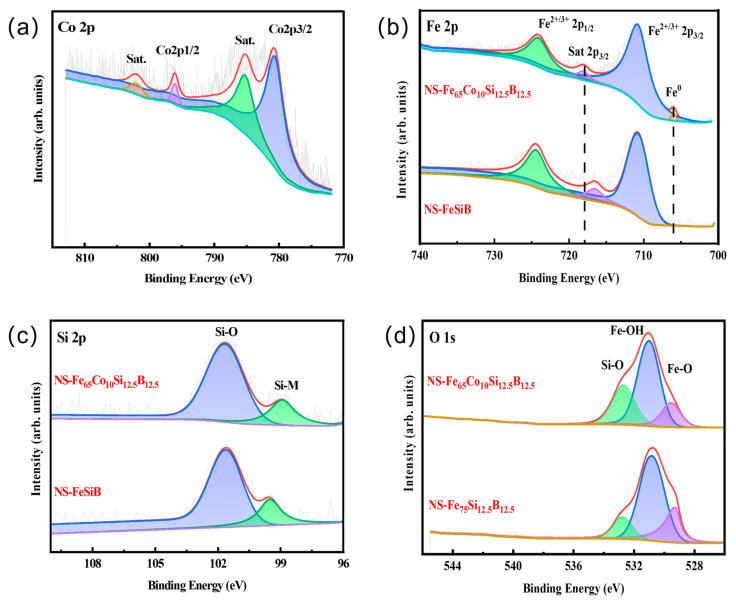
High-resolution X-ray photoelectron spectroscopy of (**a**) Co 2p, (**b**) Fe 2p, (**c**) Si 2p, and (**d**) O 1s for NS-Fe_65_Co_10_Si_12.5_B_12.5_ and Fe_65_Co_10_Si_12.5_B_12.5_.

The chemical state and doping structure of NS-Fe_65_Co_10_Si_12.5_B_12.5_ were further studied by X-ray photoelectron spectroscopy (XPS). Figure S3 shows the full spectrum of the X-ray photoelectron energy spectrum of NS-Fe_65_Co_10_Si_12.5_B_12.5_. It can be seen that Fe, Co, Si, and B are present on the surface of the sample, and the corresponding binding energy peaks are located 787 eV, 712 eV, 180 eV, and 102 eV, respectively. The energy spectrum of Co 2p is shown in Figure 3a. The two peaks near 780.78 eV and 795.98 eV correspond to the 2p3/2 and 2p1/2 peaks of the oxidation state Co [32,33], respectively. The two peaks near 785.18 eV and 802.33 eV correspond to satellite peaks of Co^2+^, respectively [24]. The satellite peak intensity of Co^2+^ ion is close to the intensity of 2p3/2, indicating that there is a large amount of Co^2+^ on the surface of NS-Fe_65_Co_10_Si_12.5_B_12.5_. Figure 3b shows the X-ray photoelectron spectra of Fe 2p. Compared with NS-FeSiB, a small amount of the Fe^0^ characteristic peak (706.03 eV) remains on the surface of NS-Fe_65_Co_10_Si_12.5_B_12.5_ after dealloying, indicating that Co doping intervention changes the valence state of Fe, and Fe atoms are more difficult to be oxidized during dealloying. By comparing the X-ray photoelectron spectra of Si 2p of the sample NS-FeSiB and sample NS-Fe_65_Co_10_Si_12.5_B_12.5_ (as shown in Figure 3c), the peak intensity of the Si and metal bond on the surface of the sample NS-Fe_65_Co_10_Si_12.5_B_12.5_ was enhanced. The results show that Co doping contributes to the bonding between Si and metal [34]. The O1s energy spectrum results (Figure 3d) show that the Si-O bond intensity is enhanced while the Fe-O bond intensity is weakened after Co doping, which is consistent with the results of Fe 2p and Si 2p.

### 2.4. Electrochemical Parameters

**Figure 4:**
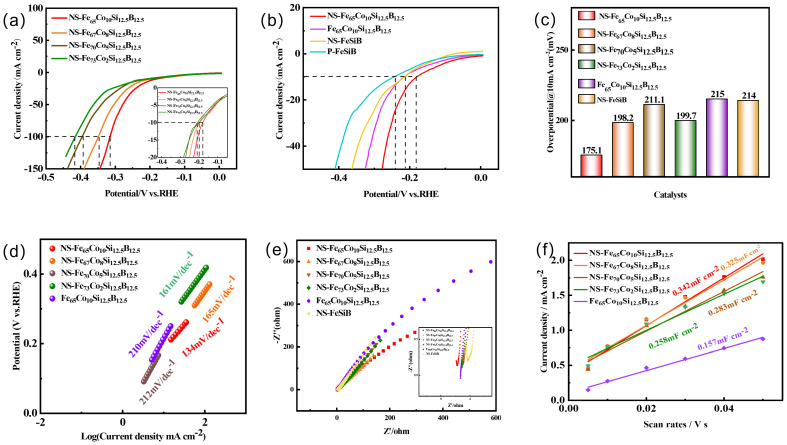
Electrochemical test data of Fe-based electrodes with different elements in 1 mol/L KOH. (**a**) Linear sweep voltammetry curves for different amounts of Co doping. (**b**) Linear sweep voltammetry curves for different samples. (**c**) Overpotentials at 10 mA cm^−2^ current density. (**d**) Tafel plots. (**e**) Nyquist plots. (**f**) Double layer capacitance at different scan rates.

Figure 4 shows the electrocatalytic performance test diagram of NS-Fe_75−x_Co_x_Si_12.5_B_12.5_ and other catalyst materials. Figure 4a shows the linear sweep voltammetry curves of different Co dopants. The overpotential of hydrogen evolution reaction decreases with the increase in Co dopants, and the addition of 10% Co has the best hydrogen evolution activity in the prepared samples. NS-Fe_65_Co_10_Si_12.5_B_12.5_ can reach 314.7 mV under the current density of 100 mA cm^−2^. It was significantly lower than NS-Fe_67_Co_8_Si_12.5_B_12.5_ (348.7 mV), NS-Fe_70_Co_5_Si_12.5_B_12.5_ (395.7 mV), and NS-Fe_73_Co_2_Si_12.5_B_12.5_ (415.7 mV). Similarly, the overpotential of NS-Fe_65_Co_10_Si_12.5_B_12.5_ at the current density of 10 mA cm^−2^ is 175.1 mV, which is attribute to the synergic effect between Fe and Co ions [35]. During the H_2_ evolution process of water splitting, the Co atom in these Co-Fe acts as the reaction site for the water O-H bond cleavage. An Fe atom can be used as the H_2_ evolution center, and the surface after corrosion has the largest electrochemically active surface area, which can expose more active sites and present a better hydrogen evolution reaction performance. As shown in Figure 4b, the comparison of the catalyst NS-Fe_65_Co_10_Si_12.5_B_12.5_ with uncorroded Fe_65_Co_10_Si_12.5_B_12.5_, NS-FeSiB, and P-FeSiB shows that the performance of FeSiB doped with Co is significantly higher than that of undoped FeSiB. The performance of uncorroded Fe_65_Co_10_Si_12.5_B_12.5_ is similar to that of the NS-FeSiB catalyst. The performance of each catalyst under a current density of 10 mA cm^−2^ is shown in Figure 4c. The overpotential of NS-Fe_65_Co_10_Si_12.5_B_12.5_, NS-Fe_67_Co_8_Si_12.5_B_12.5_, NS-Fe_70_Co_5_Si_12.5_B_12.5_, NS-Fe_73_Co_2_Si_12.5_B_12.5_, Fe_65_Co_10_Si_12.5_B_12.5_, and NS-FeSiB were 175.1 mV, 198.2 mV, 211.1 mV, 199.7 mV, 215 mV, and 214 mV, respectively.

In order to further study the effect of Co doping on FeSiB catalyst, Tafel analysis will reveal the kinetics and mechanism of the hydrogen evolution reaction [25]. As shown in Figure 4d, the Tafel slopes of NS-Fe_65_Co_10_Si_12.5_B_12.5_, NS-Fe_67_Co_8_Si_12.5_B_12.5_, NS-Fe_70_Co_5_Si_12.5_B_12.5_, NS-Fe_73_Co_2_Si_12.5_B_12.5_, and Fe_65_Co_10_Si_12.5_B_12.5_ are 134, 165, 212, 161, and 210 mV dec^−1^, respectively. These results show that the catalyst containing 10% Co is more beneficial to improve the catalytic kinetic performance of the hydrogen evolution reaction. In order to understand the charge transfer characteristics at the electrolyte/electrocatalyst interface [24,36], the AC impedance spectrum was measured, as shown in Figure 4e. It can be seen that the NS-Fe_65_Co_10_Si_12.5_B_12.5_ has the smallest charge transfer resistance, which is 2.265 Ω, smaller than NS-Fe_67_Co_8_Si_12.5_B_12.5_ (2.709 Ω), NS-Fe_70_Co_5_Si_12.5_B_12.5_ (2.961 Ω), NS-Fe_73_Co_2_Si_12.5_B_12.5_ (2.843 Ω), Fe_60_Co_10_Si_12.5_B_12.5_ (2.713 Ω), and NS-FeSiB (3.67 Ω). The results show that the electron transfer rate of 10% Co-doped FeSiB increases during hydrogen evolution, which proves that Co-doped FeSiB has superior electrocatalytic performance.

The electrochemical surface area (ECSA) value was obtained according to the equation of ECSA = C*_dl_*/C*_s_*, where C*_dl_* is the measured double-layer capacitance fitted from the CV curves, and the C*_s_* is 40 μm cm^−2^, representing a universal value of the flat and smooth specific capacitance of the electrode materials. By obtaining the slope of the line fitted by the area of the cyclic voltammetry characteristic curve and the scanning rate at different rates in the non-Faraday region, the double-layer specific capacitance *C_dl_* of different materials is obtained [37]. Figure 4f shows the linear relationship between current density difference and scanning rate when the voltage is 0.05 V in a KOH solution of 1 mol L^−1^. *C_dl_* values of NS-Fe_65_Co_10_Si_12.5_B_12.5_, NS-Fe_67_Co_8_Si_12.5_B_12.5_, NS-Fe_70_Co_5_Si_12.5_B_12.5_, NS-Fe_73_Co_2_Si_12.5_B_12.5_, and Fe_65_Co_10_Si_12.5_B_12.5_ (corresponding the ECSA values were 0.342 mF cm^−2^ (8.55), 0.325 mF cm^−2^ (8.125), 0.283 mF cm^−2^ (7.075), 0.258 mF cm^−2^ (6.45), and 0.157 mF cm^−2^ (3.925), respectively. The increase of *C*_dl_ and ECSA values is gradually increased according to the amount of Co doping, which is because Co doping improves the dispersion of the catalyst, making the catalyst particles smaller and larger than the electrochemically active surface area. Moreover, Co doping can cause electronic effects and change the electronic state of FeSiB alloy, thus affecting its catalytic performance. In addition, synergies between Co and Fe, Si and B may also lead to the formation of new active sites, increasing the electrochemically active area.

**Figure 5:**
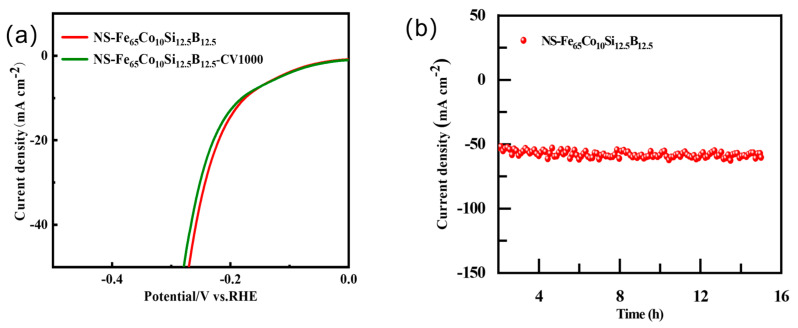
Stability for NS-Fe_65_Co_10_Si_12.5_B_12.5_. (**a**) 1000 cycles. (**b**) Stability test.

At the same time, NS-Fe_65_Co_10_Si_12.5_B_12.5_ also showed excellent hydrogen evolution stability. Figure 5 shows the stability test diagram of NS-Fe_65_Co_10_Si_12.5_B_12.5_. Figure 5a shows the comparison diagram of linear scanning voltammetry characteristic curve of NS-Fe_65_Co_10_Si_12.5_B_12.5_ before and after 1000 cycles of voltammetry. The results showed that the linear scanning voltammetry curves of NS-Fe_65_Co_10_Si_12.5_B_12.5_ did not change significantly after 1000 cycles, indicating that the hydrogen evolution reaction was stable. Figure 5b shows the change curve of catalyst current density with time at 269 mV potential. After 15 h of hydrogen evolution reaction, the current density of hydrogen evolution reaction decreased to 81.9% at the beginning, indicating that the corroded NS-Fe_65_Co_10_Si_12.5_B_12.5_ could stably undergo hydrogen evolution reaction for a long time. Co doping improves the crystallinity of the FeSiB catalyst, enhances the binding of metal bonds, and improves the lattice structure of the catalyst, which not only enhances the catalytic performance of the catalyst, but also enhances its stability.

## 3. Experimental Methods

### 3.1. Materials

Fe, FeSi, FeB, and Co raw materials (>99.9 wt.%) were purchased from Beijing Dimu Advanced Materials Co., Ltd. (Beijing, China). Potassium hydroxide (KOH AR) was provided by Shanghai Aladdin Company (Shanghai, China).

### 3.2. Preparation of Bulk Alloy Electrodes

In an electronic balance, FeB (B, 19.37%), FeSi (Si, 75%), pure Fe and Co are weighed at an atomic ratio of 75−x:12.5:12.5:x to 20 g. After that, the raw materials are put into the arc melting furnace and melted under the atmosphere of argon. High temperature is generated by the arc discharge, so that the raw materials are melted and mixed together by magnetic stirring. In the process of melting, the front and back sides are repeatedly melted five times and magnetic stirring five times. After melting, the raw materials are formed into φ6 cylindrical rods by the operation mode of arc melting and suction casting, and then cooled at room temperature. Then, the φ6 cylindrical rods are cut by a wire cutting machine into small iron sheets with a thickness of 0.5 mm and a length and width of 5 mm respectively. The cut sample is Fe_75−x_Co_x_Si_12.5_B_12.5_, and its catalyst is changed by changing the Co atomic ratio. Four kinds of Co-doped elements were prepared (Fe_75−x_Co_x_Si_12.5_B_12.5_, x = 2, 5, 8 and 10). Then, the prepared samples were put into 2 mol L^−1^ KOH solution, dealloying corrosion for 3 h, so as to obtain Fe_75−x_Co_x_Si_12.5_B_12.5_−2M−3h. It is named NS-Fe_75−x_Co_x_Si_12.5_B_12.5_.

### 3.3. Electrochemical Tests

The electrochemical performance was tested on three-electrode system in 1 mol/L KOH solution. The electrochemical workstation was CHI760E. The reference electrode, the counter electrode and the work electrode were Hg/HgO electrode, graphite rod, and the prepared samples, respectively. The RHE calibration process was performed by cyclic voltammetry characterization with an Hg/HgO (1.0 mol/L KOH) as the reference electrode and two Pt foils as the working electrode and counter electrode, respectively. The average of the voltage value at the current value of 0 mA was taken as the RHE potential. The RHE potential in this work is 0.8931 V (Figure S5). The results of electrochemical test were converted into the potential under the RHE, and the conversion formula was *E*_RHE_ = *E*_Hg/HgO_ + 0.8931 V.

The scan rate of linear sweep voltammetry curve is 5 mV/s. The electrochemical window of the cyclic voltammetry curve during the analysis of electrochemical active surface area is −0.8~−0.7 V vs. RHE (non-redox region). The scan rates of cyclic voltammetry were 5, 10, 20, 30, 40, and 50 mV s^−1^, respectively. The electrochemical impedance spectroscopy was measured at the open-circuit potential with the parameters of 0.01–100 kHz. The electrochemical HER stability was evaluated at 50 mA cm^−2^ for 15 h through chronopotentiometry.

### 3.4. Characterization

X-ray diffraction (XRD, D/max-2500) on a Rigaku diffractometer and the X-ray photoelectron spectroscopy (XPS, Thermo ESCALAB250XI, Waltham, MA, USA) were employed to investigate the phase composition, elemental composition, and valence state of the as-prepared samples. XRD used Cu Kα radiation, the diffraction angle 2θ was 10–90°, and the scan rate was 8° min^−1^. Scanning electron microscopy (SEM, Zeiss-Sigma 500, Oberkochen, Germany) was applied to investigated the morphology and structure of the samples.

## 4. Conclusions

In summary, we have successfully developed a new nanosheet Fe-based compound electrode toward high-efficiency HER in alkaline solutions by using a general dealloying method. In addition, the comparative study of HER catalyst shows that adjusting the electronic structure of Fe_75−x_Co_x_Si_12.5_B_12.5_ by the optimal doping level of Co doping can improve its HER activity in an alkaline solution. The NS-Fe_65_Co_10_Si_12.5_B_12.5_ catalyst has an overpotential of 175.1 mV and a Tafel slope of 134 mV dec^−1^ at a current density of 10 mA cm^−2^ and has a long-term stability of 15 h at a current density of 50 mA cm^−2^. This is due to the synergistic effect between Fe and Co ions in the H_2_ evolution process of electrolytic water; the Co atoms in these Co-Fe act as the reaction site for the water O-H bond cleavage, while the Fe atoms can act as the H_2_ evolution center, and the surface coating has the largest electrochemically active surface area after dealloying. Therefore, NS-Fe_65_Co_10_Si_12.5_B_12.5_ can provide the maximum active site for the electrocatalytic reaction and show the best electrocatalytic performance.

## Data Availability

Data are contained within the article and Supplementary Materials.

## Supplementary Materials

The following supporting information can be downloaded at https://www.mdpi.com/article/10.3390/molecules29174130/s1, Figure S1: (a) The SEM image of NS-Fe_65_Co_10_Si_12.5_B_12.5_ electrodes. (b) Si (c) Co and (d) Fe (e) O (f) B EDS mapping of the NS-Fe_65_Co_10_Si_12.5_B_12.5_ electrodes. Figure S2: High-resolution XPS spectra of NS-Fe_65_Co_10_Si_12.5_B_12.5_ survey. Figure S3: Measurement of double-layer capacitance of (a) Fe_73_Co_2_Si_12.5_B_12.5_ (b) Fe_70_Co_5_Si_12.5_B_12.5_ (c) Fe_67_Co_8_Si_12.5_B_12.5_ (d) Fe_65_Co_10_Si_12.5_B_12.5_ electrocatalysts in 1.0 M KOH to evaluate the electrochemically active surface area (ECSA). Figure S4: Measurement of double-layer capacitance of (a) NS-Fe_73_Co_2_Si_12.5_B_12.5_ (b) NS-Fe_70_Co_5_Si_12.5_B_12.5_ (c) NS-Fe_67_Co_8_Si_12.5_B_12.5_ (d) NS-Fe_65_Co_10_Si_12.5_B_12.5_ electrocatalysts in 1.0 M KOH to evaluate the electrochemically active surface area (ECSA). Figure S5: RHE calibration. RHE calibration of the Hg/HgO reference electrode in 1 M KOH. The calibration process was performed in high purity H2-saturated 1 M KOH with two platinum foil as working electrode and counter electrode, respectively. Hg/HgO (1 M KOH) as the reference electrode. Cyclic voltammetry (CV) was conducted at scan rate of 2 mV s^−1^, and the average of the two potentials at which the current crossed zero was taken as the thermodynamic potential for the hydrogen electrode reaction. In 1 M KOH solution, *E*_RHE_ = *E*_Hg/HgO_ + 0.8931 V.
