# Supplementary material for: Improved Alkaline Hydrogen Evolution Performance of Dealloying Fe75−xCoxSi12.5B12.5 Electrocatalyst
Source: Molecules. 2024 Aug 30;29(17):4130. doi: 10.3390/molecules29174130 (PMC11397610; doi:10.3390/molecules29174130)
Supplement: Supplementary file 1 [file molecules-29-04130-s001.zip › molecules-3177477-supplementary.pdf]

Supplementary Information for

Improved Alkaline Hydrogen Evolution Performance of Dealloying $Fe_{75-x}Co_xSi_{12.5}B_{12.5}$ Electrocatalyst

Si-Cheng Zhong[#1], Zhe Cui[#1], Jia Li[*1,2,3], Guang-Run Tian[1], Zhong-Hong Zhou[1], Hong-Fei Jiao[1], Jie-Fu Xiong[4], Li-Chen Wang[4], Jun Xiang[1], Fu-Fa Wu[*1] and Rong-Da Zhao[*1]

1 School of Materials Science and Engineering, Liaoning University of Technology, Jinzhou, Liaoning Province, 121001, People's Republic of China

2 School of Material Science and Engineering, China University of Mining and Technology, Xuzhou, Jiangsu Province, 221008, People's Republic of China

3 Innovation Center for Applied Magnetics Co., Ltd., Ningbo, Zhejiang Province, 315201, People's Republic of China

4 Ningbo Institute of Materials Technology and Engineering, Chinese Academy of Sciences, Ningbo, Zhejiang Province, 315201, People's Republic of China

E-mail: lijia4867@163.com; ffwooxy@163.com; rongdazhaoln@126.com

#These authors contributed to this work equally.

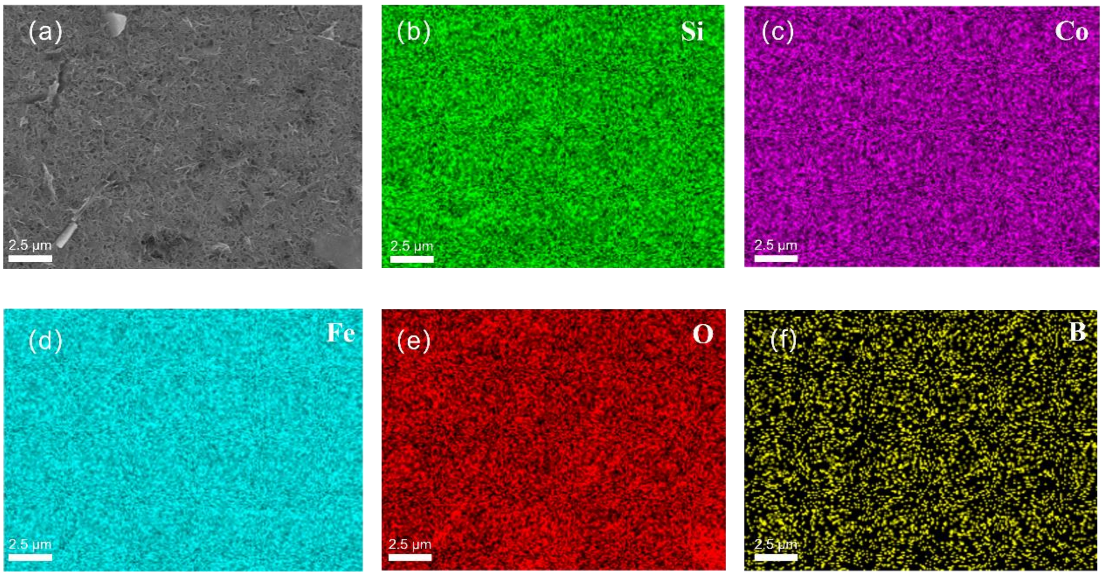

Figure S1. (a) The SEM image of NS-$Fe_{65}Co_{10}Si_{12.5}B_{12.5}$ electrodes. (b) Si (c) Co and (d) Fe (e) O (f) B EDS mapping of the NS-$Fe_{65}Co_{10}Si_{12.5}B_{12.5}$ electrodes.

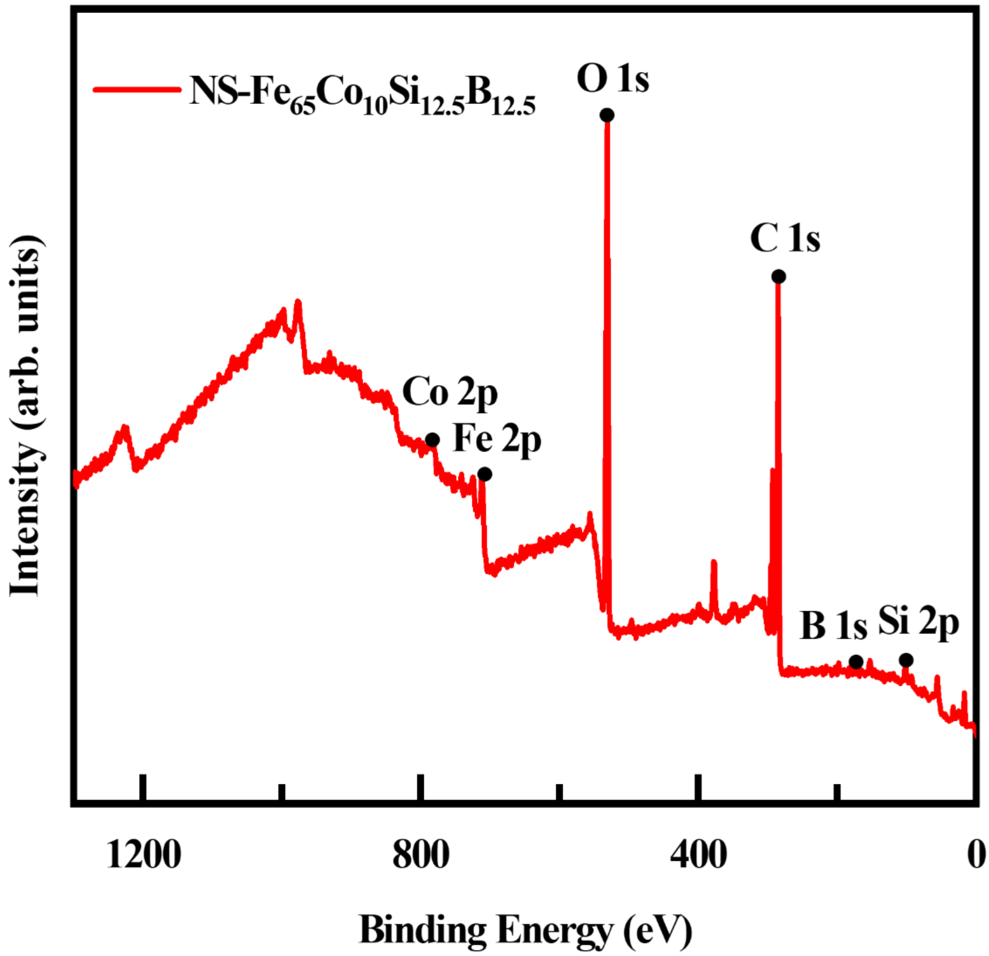

Figure S2. High-resolution XPS spectra of NS-$Fe_{65}Co_{10}Si_{12.5}B_{12.5}$ survey.

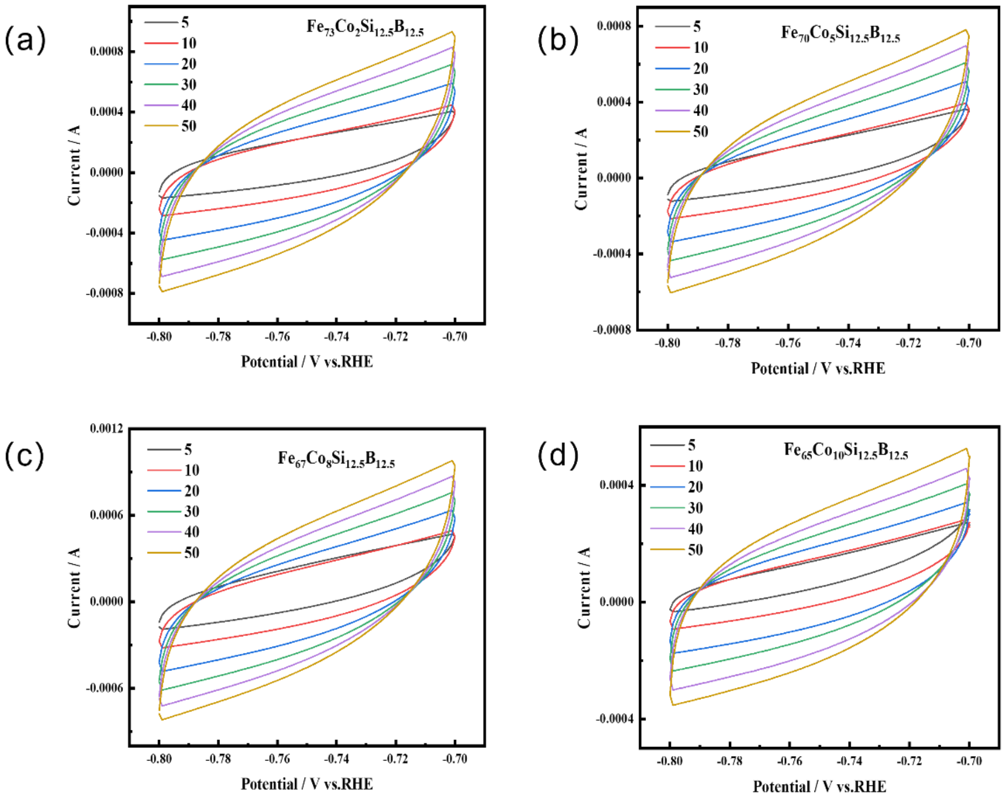

Figure S3. Measurement of double-layer capacitance of (a) $Fe_{73}Co_2Si_{12.5}B_{12.5}$ (b) $Fe_{70}Co_5Si_{12.5}B_{12.5}$ (c) $Fe_{67}Co_8Si_{12.5}B_{12.5}$ (d) $Fe_{65}Co_{10}Si_{12.5}B_{12.5}$ electrocatalysts in 1.0 M KOH to evaluate the electrochemically active surface area (ECSA).

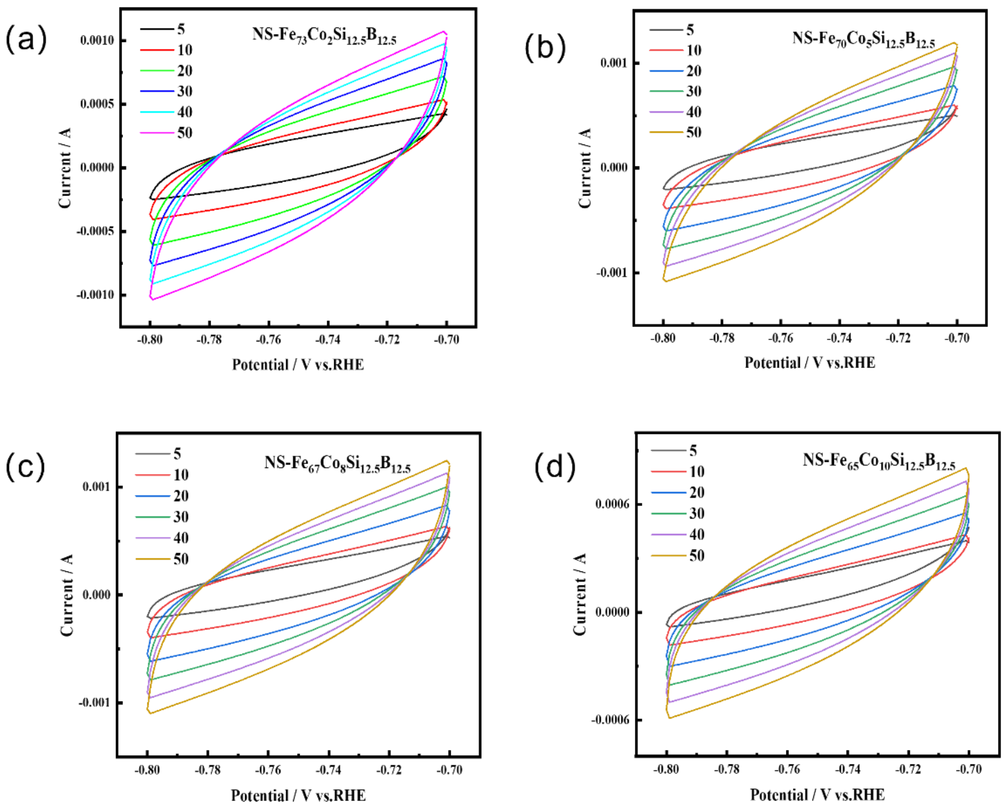

Figure S4. Measurement of double-layer capacitance of (a) NS-$Fe_{73}Co_2Si_{12.5}B_{12.5}$ (b) NS-$Fe_{70}Co_5Si_{12.5}B_{12.5}$ (c) NS-$Fe_{67}Co_8Si_{12.5}B_{12.5}$ (d) NS-$Fe_{65}Co_{10}Si_{12.5}B_{12.5}$ electrocatalysts in 1.0 M KOH to evaluate the electrochemically active surface area (ECSA).

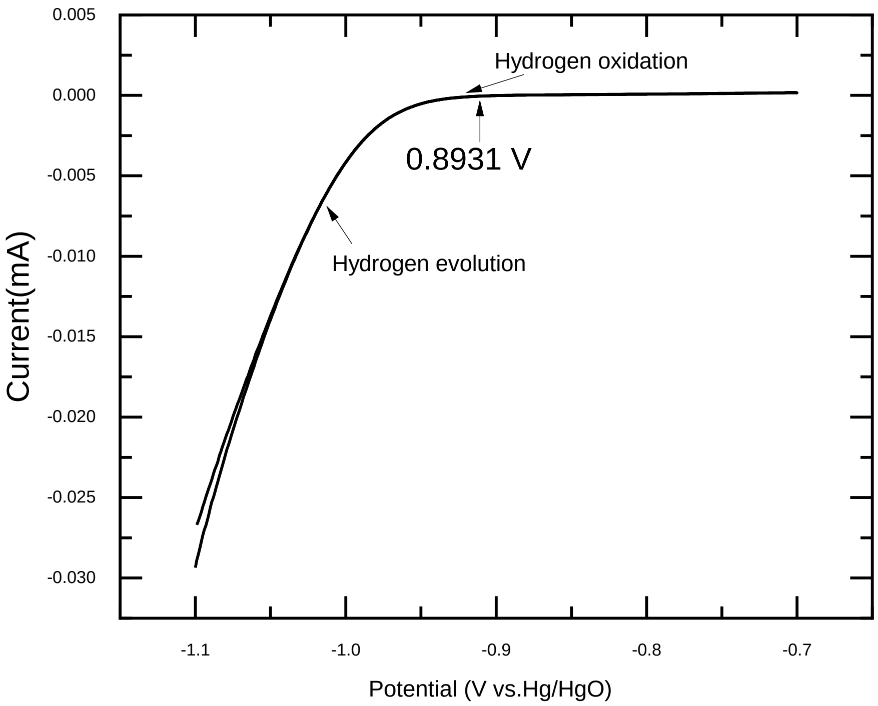

Figure S5. RHE calibration. RHE calibration of the Hg/HgO reference electrode in 1 M KOH. The calibration process was performed in high purity H_2-saturated 1 M KOH with two platinum foil as working electrode and counter electrode, respectively. Hg/HgO (1 M KOH) as the reference electrode. Cyclic voltammetry (CV) was conducted at scan rate of 2 mV s^{-1}, and the average of the two potentials at which the current crossed zero was taken as the thermodynamic potential for the hydrogen electrode reaction. In 1 M KOH solution, $E_{RHE}=E_{Hg/HgO}+0.8931V$.
